# Development and validation of an artificial intelligence based screening tool for detection of retinopathy of prematurity in a South Indian population

**DOI:** 10.3389/fped.2023.1197237

**Published:** 2023-09-19

**Authors:** Divya Parthasarathy Rao, Florian M. Savoy, Joshua Zhi En Tan, Brian Pei-En Fung, Chiran Mandula Bopitiya, Anand Sivaraman, Anand Vinekar

**Affiliations:** ^1^ Artificial Intelligence Research and Development, Remidio Innovative Solutions Inc., Glen Allen, VA, United States; ^2^ Artificial Intelligence Research and Development, Medios Technologies Pvt. Ltd., Singapore, Singapore; ^3^ Artificial Intelligence Research and Development, Remidio Innovative Solutions Pvt. Ltd., Bangalore, India; ^4^ Department of Pediatric Retina, Narayana Nethralaya Eye Institute, Bangalore, India

**Keywords:** retinopathy of prematurity, artificial intelligence, screening, deep learning, accessibility, ROP, infant blindness, AI

## Abstract

**Purpose:**

The primary objective of this study was to develop and validate an AI algorithm as a screening tool for the detection of retinopathy of prematurity (ROP).

**Participants:**

Images were collected from infants enrolled in the KIDROP tele-ROP screening program.

**Methods:**

We developed a deep learning (DL) algorithm with 227,326 wide-field images from multiple camera systems obtained from the KIDROP tele-ROP screening program in India over an 11-year period. 37,477 temporal retina images were utilized with the dataset split into train (*n* = 25,982, 69.33%), validation (*n* = 4,006, 10.69%), and an independent test set (*n* = 7,489, 19.98%). The algorithm consists of a binary classifier that distinguishes between the presence of ROP (Stages 1–3) and the absence of ROP. The image labels were retrieved from the daily registers of the tele-ROP program. They consist of per-eye diagnoses provided by trained ROP graders based on all images captured during the screening session. Infants requiring treatment and a proportion of those not requiring urgent referral had an additional confirmatory diagnosis from an ROP specialist.

**Results:**

Of the 7,489 temporal images analyzed in the test set, 2,249 (30.0%) images showed the presence of ROP. The sensitivity and specificity to detect ROP was 91.46% (95% CI: 90.23%–92.59%) and 91.22% (95% CI: 90.42%–91.97%), respectively, while the positive predictive value (PPV) was 81.72% (95% CI: 80.37%–83.00%), negative predictive value (NPV) was 96.14% (95% CI: 95.60%–96.61%) and the AUROC was 0.970.

**Conclusion:**

The novel ROP screening algorithm demonstrated high sensitivity and specificity in detecting the presence of ROP. A prospective clinical validation in a real-world tele-ROP platform is under consideration. It has the potential to lower the number of screening sessions required to be conducted by a specialist for a high-risk preterm infant thus significantly improving workflow efficiency.

## Introduction

Retinopathy of prematurity (ROP) is one of the leading causes of infant blindness worldwide. It is a disease of the retina that affects low birth weight premature infants, which can lead to irreversible blindness if left untreated. Improving healthcare systems in low to middle-income countries has led to better survival rates for babies born prematurely. However, without timely diagnosis and treatment such infants are at risk for developing ROP due to their low birth weight. A lack of experts in the field of ophthalmology combined with improved survival rates of premature babies, therefore, make ROP a major public health problem (1–4). India has 3.5 million premature babies born annually, the highest number anywhere in the world. The numbers of infants with ROP are on the rise (5). The potential at-risk population of ROP is understood as babies born under 2,000 grams. This concerns around 9% of births in India (6).

Traditionally, ROP screening is done by a pediatric vitreo-retina specialist using a bedside clinical exam. This is available primarily in large cities (7). ROP screening programs are currently grossly inadequate, particularly in remote settings due to the insufficient doctor-population ratio, with less than 200 ROP specialists in India (8, 9). As a result, babies at risk for ROP are often unable to get the necessary screening and diagnosis in a timely manner. This is particularly true in places where medical resources are scarce such as in small rural centers. ROP is highly treatable with early screening and regular monitoring (10). In recent years, the adoption of widefield retinal imaging has enabled tele-ROP screening. This is done in collaboration with a reading center providing the diagnosis (11). In India, this approach has been extensively used to screen in rural areas (12–16). While this has been a significant advancement, there remains a large unscreened high-risk population. Furthermore, ROP diagnosis can be inconsistent (17, 18). For premature infants, such variability leads to clinically significant differences in outcomes (19). An objective tool is therefore necessary. It can help standardize care irrespective of the location where the infant is being managed. A combination of the benefits of wide-field imaging and automated diagnosis integrated on a teleophthalmology platform can solve these issues. Such a model can enable rapid screening and triaging of infants even in a low-resource setting.

Artificial intelligence methodologies have been used to improve the diagnosis of many medical conditions, including retinopathy of prematurity. Deep learning (DL) consists of computer-based analysis systems trained to automatically recognize and evaluate images or other inputs (20). It has been successfully used to diagnose a variety of ocular conditions, most notably diabetic retinopathy (21). Recently, quite a few studies have been published on the use of DL to screen for plus disease in ROP (4, 22–33). However, as a first step, there is a need for an automated tool that can screen the huge at-risk ROP population in India. This led us to develop an AI model that provides a binary output for the presence or absence of ROP.

In this study, we have trained a DL algorithm using a substantial dataset of temporal images that were captured with various wide-field imaging systems from the largest single-hospital tele-ROP screening program in the world. The development of the DL algorithm for automatic detection of ROP in retinal images of premature babies was implemented using a binary classification model (ROP present vs. ROP absent). Further, we compare the accuracy of the DL method on an independent test set with the diagnosis from trained ROP graders and ROP specialists.

## Methods

The study adhered to the tenets of the Declaration of Helsinki and was approved by the Institutional Ethical Committee of Narayana Nethralaya, a tertiary eye hospital in South India. For algorithm development, a total of 227,326 anonymized images from 5,944 premature infants who underwent ROP screening were retrospectively collected and included following a written informed consent. They were captured using wide-field RetCam cameras (RetCam III, RetCam shuttle, Natus technologies, Middleton, USA) or Forus (3netra NEO, Forus Health Pvt Ltd, Bengaluru, India) in the KIDROP tele-ROP screening program over an 11 year period (from 2011 to 2022). The tele-ROP program covers 30 districts across 4 zones in South India. This encompasses 124 Neonatal centers, level 2 and Level 3, that cover special newborn care units (SNCUs) managed by the Govt as well as NICUs that are part of tertiary centers. The imaging protocol has been described in detail elsewhere. In brief, on each eye of an infant, a minimum of six fundus images were obtained using the 130-degree lens provided by the manufacturer and included- macula center, disc center, temporal, superior, nasal and inferior quadrants (12). Follow-up images from infants who underwent additional screenings were included. An assistive neural network was used for filtering temporal images from other views. 37,477 temporal images of no ROP and varying severities of ROP as outlined below (Table 1) were utilized for training and testing the binary classifier. These included images of varying image quality to develop a robust model that could also perform well on par quality images.

**Table 1:** Dataset split by stages.

	Train	Validation	Test
No ROP	17,929	2,698	5,240
Stage 1 ROP	1,568	207	398
Stage 2 ROP	5,919	923	1,609
Stage 3 ROP	566	178	242
Total	25,982	4,006	7,489
Camera split			
RetCam	20,536	3,075	5,783
NEO	5,446	931	1,706

### Image labeling

**Figure 1:**
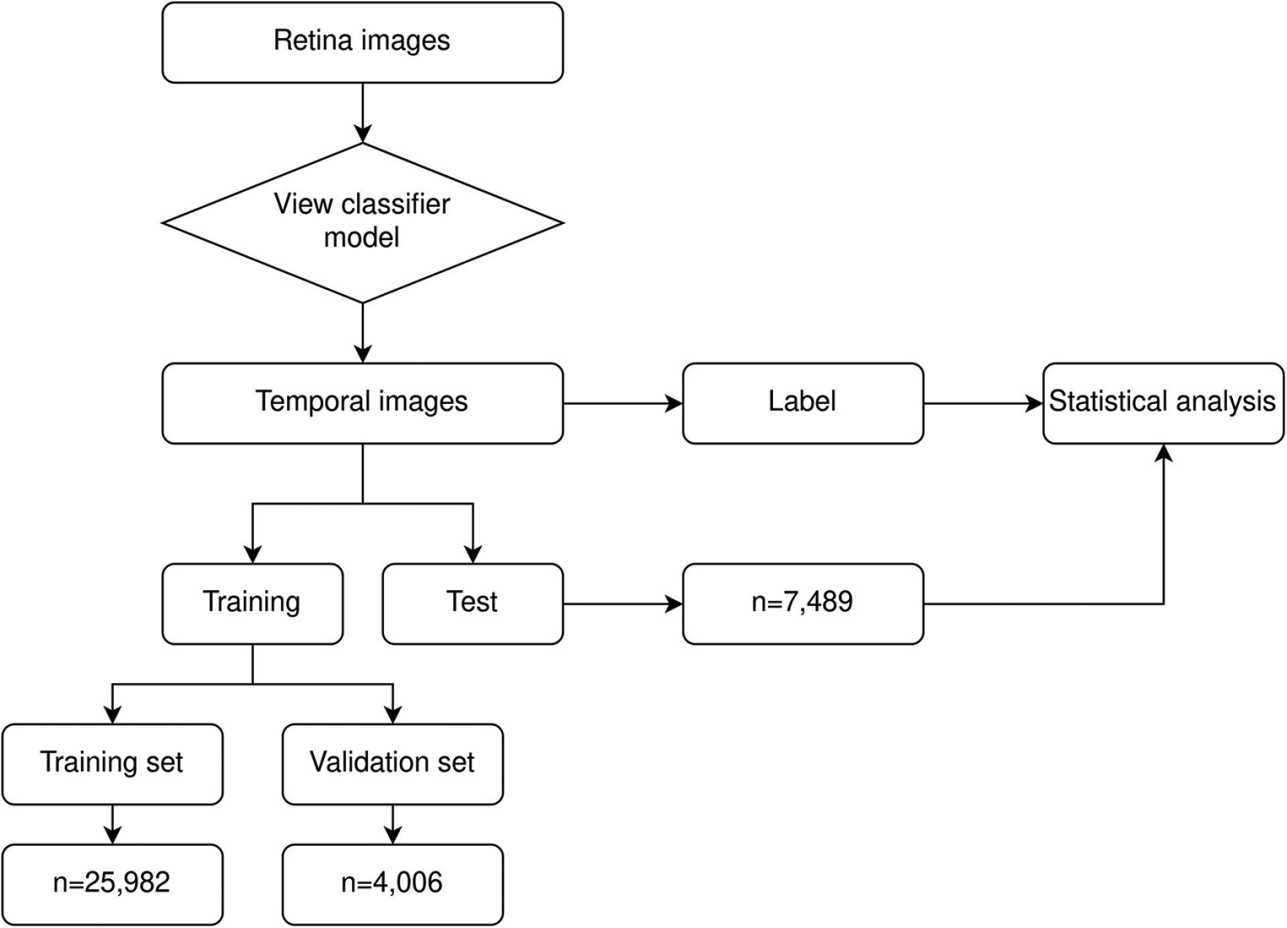
Experimental design.

The experimental design is shown in the flowchart, Figure 1 below. The retinal images used for development were labeled by trained ROP graders (Level I to Level III) as part of the tele-ROP program. The robust training, validation, and accreditation of the technicians has been described previously (12). In brief, when compared to an ROP specialist for any stage of ROP, a Level I technician has a sensitivity and specificity of 94.5% (95% CI: 92.9%–95.9%) and 84% (95% CI: 92.9%–95.9%), and Level III has a sensitivity and specificity of 99.4% (95% CI: 97.7%–99.9%) and 93.2% (95% CI: 87.8%–96.7%) respectively. Level I technicians agreed with 85.9% of the experts and when comparing “treatment vs. no treatment”, “mild vs. severe ROP” and “discharge vs. no discharge” with ROP specialists, the Kappa (agreement) scores are 0.63, 0.61, and 0.79 respectively. Level III technicians agreed with 94.3% of the experts. When comparing “treatment vs. no treatment”, “mild vs. severe ROP” and “discharge vs. no discharge” with ROP specialists, the Kappa (agreement) scores are 0.85, 0.84, and 0.94 respectively. A Level I technician would miss 0.9% of infants needing treatment and a Level III only 0.4%. Based on the color coded decision aiding algorithm utilized in the KIDROP program, all premature infants with urgent/treatment warranted ROP (“red” triage; Stage 2 with no plus in non-zone 1 location, plus or pre plus with any stage any zone, zone 1 disease any stage, aggressive posterior ROP), 10%–15% of those requiring follow-up (“orange” triage; Stage 1 ROP, no plus in non-zone 1 location, immature retina, regressing ROP), and a small proportion of those being discharged from the screening program (“green” triage; mature blood vessels) are ratified by an ROP expert by images being reviewed on the live tele-ROP platform or by clinical examination on site wherever possible. The graders/experts followed the International Clinical Classification of Retinopathy of Prematurity (ICROP 2 or ICROP 3 as relevant for the period of image acquisition). The diagnoses were retrieved from the daily registers of the program. Per-eye diagnoses were based on all images captured during the screening session. The per-eye diagnosis was merged into two categories: ROP absent (“no ROP”) and ROP present (ROP stage 1 to stage 3).

### AI development

We used convolutional neural networks (CNNs), a class of deep learning models, to train the classifier (Medios AI-ROP, Medios Technologies, Remidio Innovative Solutions, Singapore). In particular, we used the EfficientNet-B0 architecture. The EfficientNet family of models improve on the previous generation of CNNs by introducing a new scaling method that balances network depth, width, and resolution. Furthermore, the baseline architecture was designed using neural architecture search. This consists of an automated process optimizing network architecture for complexity and accuracy. These developments allow the EfficientNet architecture to have the same modeling capabilities while having fewer parameters compared to other architectures. Within the EfficientNet family, we chose the EfficientNet-B0 architecture, which is the least demanding in terms of memory and computational power. This choice of architecture opens the door to efficient and offline deployment of the model, with results obtained within a fraction of a second.

The model has been initialized as a binary classifier and has learned to distinguish temporal fundus images of no ROP from those of images with ROP of stages 1–3. We chose this model of training since an architecture taking a single image as input is less complex and easier to train than an architecture with multiple input images. Furthermore, a temporal image is sufficient for a binary detection of the presence of ROP in a majority of cases. The dataset was split into a train, validation, and test set. The breakdown of each split by camera system and ROP stages is detailed in Table 1. The train and validation sets were used throughout the training process, while the test set was used for an independent and final evaluation of the model. Furthermore, the training set has been constructed to ensure that there is no patient overlap between the validation and test set. This is to ensure that there is no data leakage when training.

**Figure 2:**
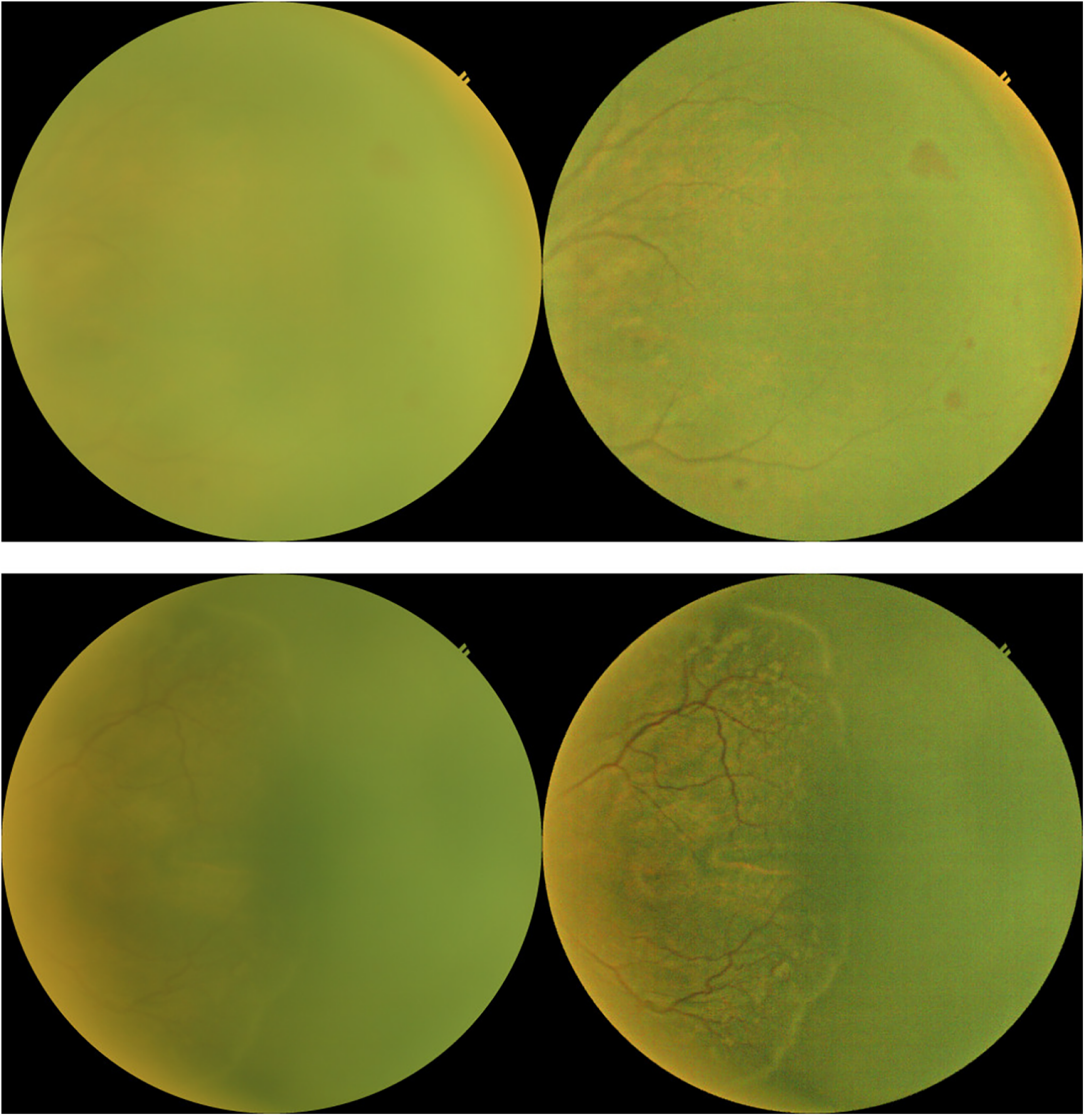
Example of images before (left images) and after image processing (right images).

We used a model pre-trained on ImageNet, a large and generic data classification task. This is a training method called transfer learning, which has been shown to produce faster convergence and higher accuracies. An image processing method was designed to enhance the contrast of input images. The method relies on bilateral filtering. Figure 2 shows an example of images before and after the image processing step. Furthermore, images of left eyes were horizontally flipped to appear like right eye images. Data augmentation techniques were limited to slight, random rotations. The model outputs a probability of presence of ROP, on top of which a threshold is applied. Different thresholds lead to different compromises between sensitivity and specificity. Class activation mapping was also implemented. This serves as a visual check with the class activation mapping highlighting the areas of the ROP image that might have triggered a positive diagnosis. Physicians can validate the quality of the model by comparing if the highlighted areas by the model indeed correspond to a positive class from their diagnosis. Anecdotal assessment of the model's activation maps has shown that it is relying on ROP features such as the ridge to form a diagnosis. The model was trained with Keras and Tensorflow on the Azure ML cloud platform.

### Statistical analysis

The performance of the algorithm was evaluated on an independent test data set by comparing it against human graders. The ground truth for these images were those provided by trained ROP graders/experts as illustrated above in the methodology. Performance was assessed by sensitivity, specificity, positive and negative predictive values (PPV, NPV). The receiver operating characteristic (ROC) analysis and area under curve (AUC) with 95% CIs were also calculated. Statistical analyses were performed using the Pandas data science library in the Python programming language.

## Results

**Figure 3:**
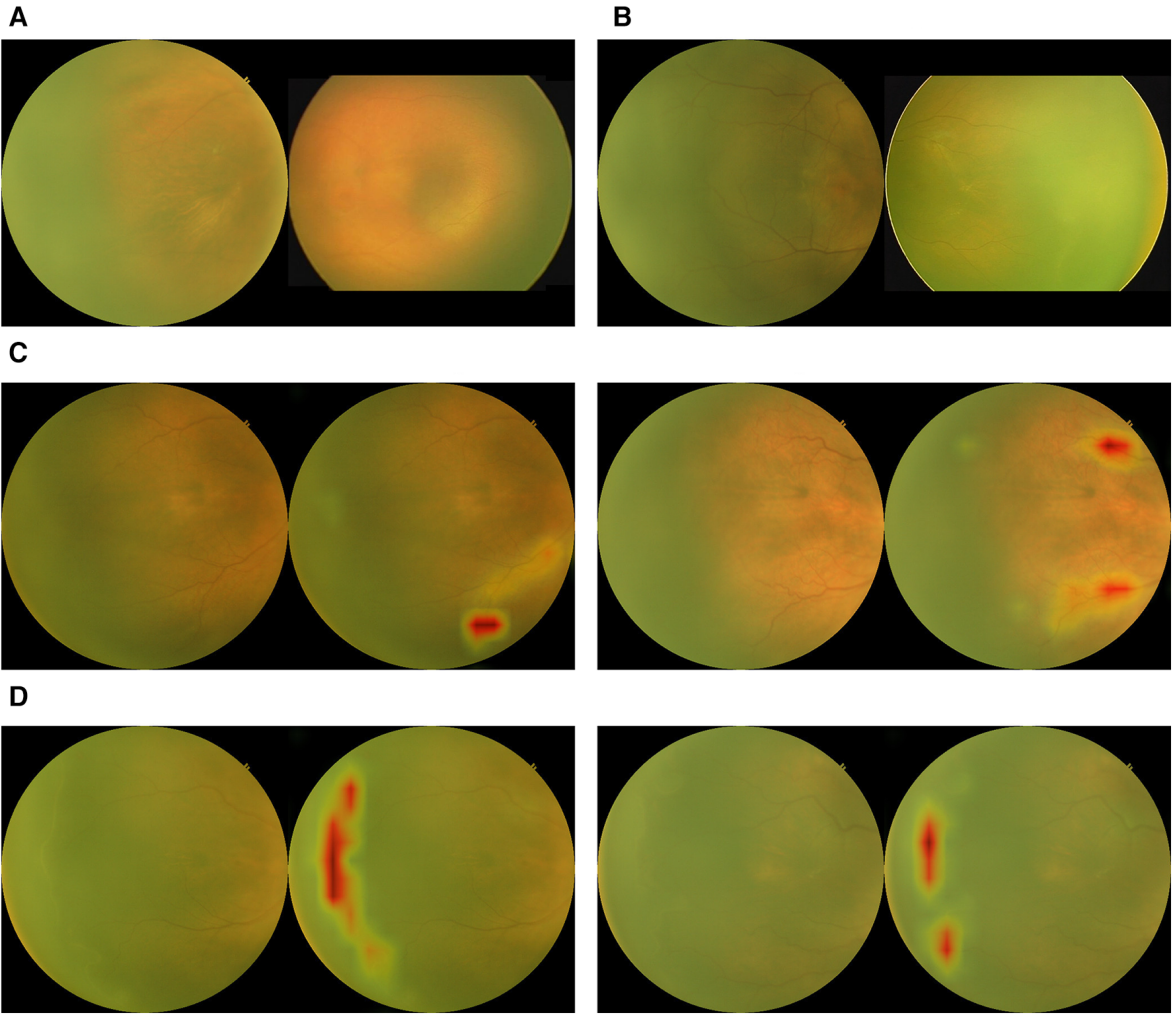
Examples of outputs from the binary classification algorithm with activation maps. (**A**) True negatives. (**B**) False negatives. (**C**) False positives with activation maps. (**D**) True positives with activation maps.

**Figure 4:**
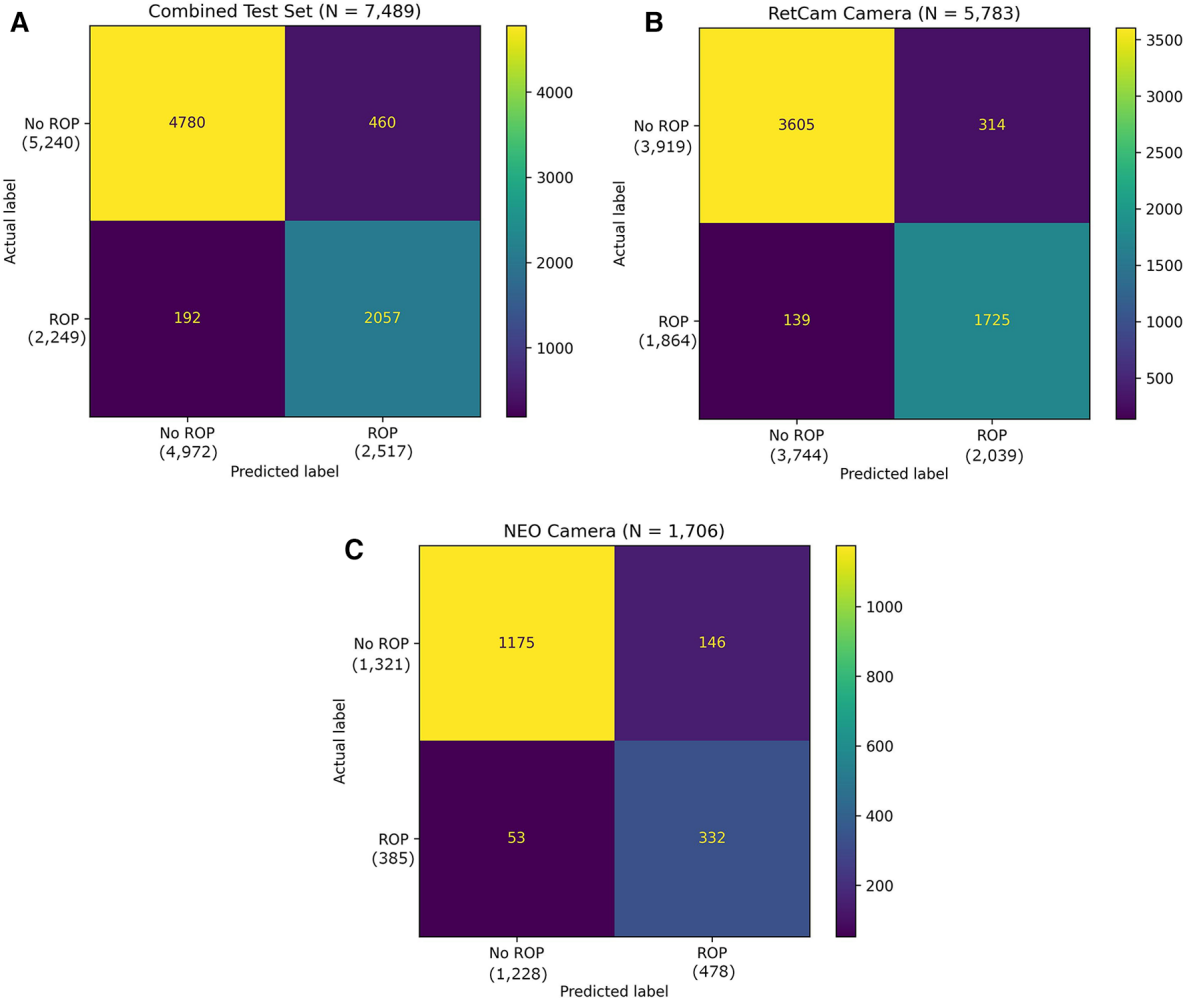
(**A**) Confusion matrix of the binary classifier on combined test dataset. (**B**) Confusion matrix of the binary classifier on test dataset with RetCam Camera. (**C**) Confusion matrix of the binary classifier on test dataset with NEO Camera.

The test set consisted of 7,489 temporal images from RetCam systems (RetCam III and RetCam Shuttle) and 3netra Neo with 2,249 (30.0%) images of varying severity of ROP. The AI analysis on a single temporal image was compared against an eye-level diagnosis (from 6 different views) made by human graders and specialists. Examples of images analyzed by the algorithm are shown in Figure 3. Figure 4A shows the performance of the algorithm on the overall test dataset as a confusion matrix.

**Figure 5:**
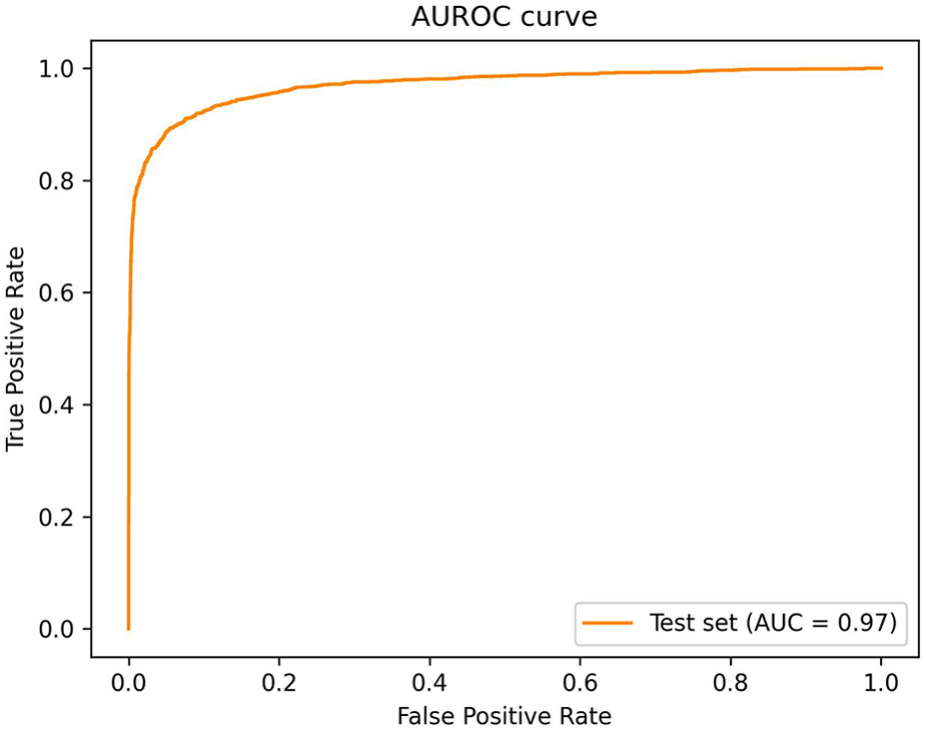
Area under the curve for ROP (stage 1 and above).

The overall sensitivity and specificity of the AI algorithm in detecting ROP were 91.46% (95% CI: 90.23%–92.59%) and 91.22% (95% CI: 90.42%–91.97%), respectively (Table 2). The PPV was 81.72% (95% CI: 80.37%–83.00%) and NPV was 96.14% (95% CI: 95.60%–96.61%). The AUROC was 0.9701 (Figure 5). 460 images classified as “ROP” by the AI were false positives. There were 192 false negatives, 97 of which belonged to Stage 1, 83 belonged to Stage 2 and 12 belonged to Stage 3. Out of the 192 false negative images, 136 (70.9%) came from a patient with another AI positive temporal image captured during the same visit with a better image quality, 38 (19.8%) had another AI positive temporal image captured during subsequent visits while 18 (9.4%) had no other AI positive temporal image. The performance breakdown of the AI algorithm by camera systems within the test as a confusion matrix is provided in Figures 4B,C.

**Table 2:** Performance metrics of AI algorithm on test set (95% CI).

	RetCam (*N* = 5,783)	NEO (*N* = 1,706)	Combined (*N* = 7,489)
Sensitivity	92.54% (91.26%–93.69%)	86.23% (82.38%–89.52%)	91.46% (90.23%–92.59%)
Specificity	91.99% (91.09%–92.82%)	88.95% (87.13%–90.59%)	91.22% (90.42%–91.97%)
Accuracy	92.17% (91.44%–92.85%)	88.34% (86.72%–89.82%)	91.29% (90.63%–91.92%)

## Discussion

We developed a novel DL-based binary classification model for ROP detection using a large dataset from a tele-ROP screening program in South India. This algorithm exhibited promising performance with a sensitivity of 91.46% and specificity of 91.22% across multiple camera systems. This was comparable to manual grading by trained ROP graders. In addition to image classification, the automated system can highlight areas of abnormality triggering the AI diagnosis through activation maps. We limited this model to a binary classification task. As a first step, the model is only indicating the presence of ROP without distinguishing between stages. We have demonstrated that this strategy was key in achieving good accuracy using temporal images only. Using an efficient network architecture, transfer learning as a training strategy, and a robust ground truth, the proposed system performed effectively while being computationally efficient. This paves the way for an offline, on-the-edge deployment of the model that would not require internet connectivity to make an inference. This would be a significant contribution to ROP management in low-resource settings and rural outreach such as those covered by the KIDROP program. It has the potential to reduce the number of screening sessions to be conducted by a specialist for a high-risk preterm infant thus significantly improving workflow efficiency. Fewer visits to the screening center from villages and taluks placed geographically distant from the district headquarters could potentially reduce the morbidity of these premature and often sick infants.

Cogan and Patz through their work have shown that at 8 months of embryonic life, only the nasal retinal periphery is fully vascularized (34, 35). The temporal retinal periphery is not completely vascularized until shortly after full-term birth of an infant. Since the larger temporal periphery is the last part of the retina to become fully vascularized, this anatomical area has a substantially larger ischemic area than the nasal retina. This vascularization pattern of the retina explains the greater susceptibility of the temporal retina to ROP. Hence, this is the most probable location for the disease to initiate in premature infants over 28 weeks (representing >98% of our cohort) and hence one of our rationales for using temporal quadrant images for training. This premise is also supported by Fielder et al. (36) who described the natural history of ROP with respect to the site (quadrant) of the disease and showed that the disease was visible more in the horizontal retinal regions and spared the vertical (superior and inferior) quadrants. Additionally, it has been shown that the temporal retinal disease status in ROP is strongly predictive and a reliable indicator of the disease status of the entire retina (37). Being far easier to image than the nasal periphery, image quality tends to be superior as well. This forms the scientific basis of our choice of temporal images to simplify the development process of this binary model to detect the presence or absence of ROP. However, the idea is to eventually combine this screening model with a treatment-triaging model (plus disease/type 1 & 2 disease detection model) that analyzes other views to give a more comprehensive prediction of the presence and severity of ROP and our results with the temporal retina are both encouraging and provide a platform for further enhancement studies in the real world.

It may be argued that in the interim if universal image sets from other population cohorts are validated through the model, ROP that can occur in the nasal retina, especially for extremely premature babies may be potentially missed. While we recognize this as a limitation, the natural history described by Fielder et al. (36) in 1992, reported that those who showed a nasal first disease were born more premature (GA), and almost all (25/27 infants) eventually developed stage 3 (and involved both nasal and temporal quadrants). The last two infants did not develop stage 3. It is safe, therefore, to assume that, given our current cohort, a “severe” early “nasal only” image will be in zone 1 and/or will develop temporal disease “as well” in a subsequent image, most certainly by the time the disease has reached Type 1 ROP that merits treatment. Similarly, more recently in 2010, although the Swedish group (Austeng D et al) reported nasal “onset” of disease in 27% of eyes in babies born extremely premature, it must be noted that all these cases eventually had the temporal disease in the subsequent visit (38). Furthermore, it is of significance that no baby who required treatment had only nasal disease. Even if it is assumed that nasal disease will be missed by our current AI algorithm because of not being trained on nasal-only images, the disease would be picked up in a subsequent visit on the temporal image. Our population is considerably older in gestational age than Western cohorts, and hence the current algorithm covers such an eventuality. We are encouraged to include nasal images with and without disease to test this hypothesis in future studies.

The development of this algorithm on a single temporal field of view does not limit us from running the binary algorithm on other fields of view. Given that ROP stages remain the same irrespective of the field of view, the algorithm will pick up ROP changes in any field. The final deployment strategy on temporal image alone vs. temporal image with an additional field of view will be taken based on real-world evidence and will be adjusted accordingly. The idea of using the least number of images in an AI algorithm is to simplify the tele-ROP program. In our setting, non-physicians and certified imagers capture these images (12). It is infinitely easier to capture temporal images, where in our cohort, nearly all babies who have the disease will display the same. An algorithm that simplifies the capture and assessment would serve better in a holistic tele-ROP program, making it more scalable to settings that have fewer ROP specialists and non-physicians trained to image.

15 million of the total 115 million yearly births globally are delivered prematurely (5). The highest burden falls on middle-income countries (8.2 M), followed by low-income (5.6 M) and high-income countries (1.2 M). These high-risk premature infants need on average 3–4 screening sessions before follow-up can be discontinued. This poses an additional burden on ROP specialists in resource-limited settings. An AI that can screen for the presence of disease becomes imperative and can significantly reduce the number of screening sessions required. This can also help decentralize care, moving triaging away from an ophthalmologist-led system. It can empower other caregivers like nurses in Neonatal Intensive Care Units. This will improve screening coverage and add objectivity to diagnosis. The specialist can then focus on providing treatment for complex cases.

Computer-based feature extraction tools for automated diagnosis of ROP such as Retinal image multiScale Analysis (RISA) (39), ROPTool (40), VesselMa (41) have been around since the mid-2000s. These tools use features such as vessel dilation or tortuosity as variables to diagnose plus disease. In recent years, advances in DL have led to the development of several algorithms for fully automated detection of ROP. Most prior studies have used machine learning to detect plus disease with encouraging results (22, 24, 25, 42, 43). Plus disease is the treatment requiring form of ROP as per the ICROP. It is important to identify, but it is not sufficient to define ROP itself. To the best of our knowledge, there has been very little work focused on ROP identification with the potential for an offline capability that is essential in most developing nations. Our AI algorithm stands to add tremendous value as a base algorithm for screening in high-burden countries and has the potential to reduce the number of screening sessions needed.

Wang et al. had trained an automated ROP detection system (DeepROP) using deep neural networks (DNNs) for ROP identification and grading. They used two specific DNN models exhibiting high sensitivity and specificity values (44). Training dataset consisted of a similar number of images to ours. This system, however, uses multiple images from all fields of view from an eye as input that can make deployment in the real-world far more complex. This is done through a feature binding block in the neural network architectures. On a Chinese test dataset, the identification algorithm exhibited a remarkable sensitivity and specificity of 96.2% and 99.3%, respectively, better than our model on Indian eyes. This could potentially be attributed to using highly curated images that were of superior quality for model development and testing. Furthermore, when implemented in a clinical setting, their ROP identification model had a much lower sensitivity of 84.91% and a specificity of 96.09%, highlighting the importance of real-world evidence (44). Their model additionally provides staging of disease. The advantage of our model is that it uses only temporal images to provide a binary classification with sufficient enough accuracy as a screening tool. We prioritized real-world deployment and model efficiency by choosing a less complex and shallow model EfficientNetB0 and only used the temporal view of the eye during development. In doing so, we ensure that deployment (where images are captured by non-physician technicians) is more feasible since capturing a single view of the eye is less operationally demanding than requiring all views. An interesting point to note is that while temporal-only images were used during development, the testing was by comparing against an eye-level diagnosis made with 6 different views by human experts and not against a temporal image-level diagnosis. Thus, the AI was challenged to make a prediction at an eye-level with a single temporal image precluding any selection bias during testing. This allowed for a true interpretation of the model's performance.

Tong et al. developed an automated feature-learning approach for ROP detection using DL methods displaying a sensitivity of 77.8% and specificity of 93.2% (45). In addition to image classification, the system could accurately identify the stage of ROP and the presence of plus disease. They used a similar transfer learning approach with comparable dataset size. However, the accuracy was reported to be lower than ours with a larger number of false negative cases. Vijayalakshmi et al. developed an automated detection and classification of ROP images using Hessian analysis and a support vector machine. They achieved a sensitivity of 90.37% and a much lower specificity of 64.65% (46). They however relied on a small dataset of 160 images, comprising no ROP, stage 2 and stage 3 ROP only. Hu et al. used a smaller dataset of 3,074 images and developed a screening model that achieved impressive results with a sensitivity of 96%, specificity of 98%, and AUROC of 0.992 (47). However, all of these models have a significant limitation of relying on large model architectures. This translates to requiring either a cloud server or a high computational power machine for deployment. While direct comparison with other groups is challenging due to differences in the dataset, disease distribution, ground truth diagnosis, and type of model, our results were comparable with an advantage in the model architecture used facilitating easy offline deployment on multiple camera systems (Table 3).

**Table 3:** Performance of AI algorithms in the detection of ROP.

Authors	#images	Model	Labels	Sensitivity % (95% CI)	Specificity % (95% CI)	AUROC
Present study	227,326	CNN/EfficientNet	No ROP/ROP	91.46 (90.23–92.59)	91.22 (90.42–91.97)	97.01
Wang et al. (34)	20,795	DNN/Id-Net	normal/ROP (minor/severe)	96.2 (92.29–98.89)	99.3 (96.29–99.89)	99.49
Tong et al. (35)	36,231	CNN/ResNet	Normal/mild/urgent/semi-urgent	77.8 (−)	93.2 (−)	–
Vijayalakshmi et al. (36)	160	Hessian analysis/SVM	Normal/ROP	90.37 (−)	64.65 (−)	–
Hu et al. (37)	2,668	CNN/ImageNet	Normal/ROP (mild/severe)	96 (−)	98 (−)	99.22

DNN, deep neural network; CNN, convoluted neural network; SVM, support vector machine.

There is currently no regulatory-approved AI for ROP and hence no pre-specified sensitivity and specificity performance endpoints. For referable diabetic retinopathy, the FDA-mandated superiority endpoints were 85% sensitivity and 82.5% specificity (48). We set the benchmark for the developed algorithm at 93% sensitivity and 85% specificity. These numbers match the high bar set during training for the Level 1 Graders in the KIDROP program (12). We acknowledge that while overall results (combined camera systems) and RetCam results are good, the NEO camera results can be optimized further. The results on the NEO camera can be improved by increasing the training dataset coming from this specific camera system.

A breakdown by stages of the results indicated that the false negatives were predominantly in Stage 1 ROP zone 3. In this scenario, feature identification is tricky not only for the AI but also for trained ROP graders. The results also indicate that the developed algorithm holds great potential for identifying moderate to severe cases of ROP with higher accuracy. National screening guidelines require the infant to be closely monitored over a few weeks. This strategy mitigates the risk of false negatives, for example by ensuring two consecutive normal reports by the AI. As seen in this study, 20% of false negative images were flagged positive in subsequent follow-up visits of the infant. Additionally, ensuring a visit after 40 weeks post-menstrual age (PMA) will mitigate this risk further. This is similar to the approach used in the KIDROP model. Trained technicians categorize infants with no ROP in the “green” code as per the triaging model after 40 weeks PMA only (12). These infants would then be discharged from ROP screening. This strategy will provide additional opportunities to screen the infant with the AI before declaring the infant to be normal.

The strengths of the study are the large dataset forming the backbone of the robust development process. The dataset is representative of the population it is intended to be used on and has a good distribution of disease severity and image quality, along with a robust ground truth. Having images from multiple camera systems brings about the potential for a device agnostic algorithm, allowing it to be more adaptable. A robust algorithm that can cater to various camera systems will help with usability in various real world clinical settings. Additionally, using lightweight models without compromising on performance paves the way for an on-the-edge, efficient deployment. The use of a large independent test dataset, unseen during model training, reflects the promise this model holds.

This study has three main limitations. First, the dataset comes only from a South Asian population. This may limit the generalizability of the current model. It will require an expansion in the diversity of the current dataset for deployment beyond this population. Second, ungradable image quality can contribute to misdiagnosis. While variable image quality added to the robustness of the model, an algorithm that can filter for minimum image quality and alert the operator is necessary which is currently underway. Finally, the performance of the algorithm on stages 4 & 5 disease is not clear due to inadequate representation of these very advanced stages. We are also working towards combining this screening model with a triaging model that detects treatment requiring ROP that will provide a more holistic solution. Further, real-world testing with a diverse population is necessary prior to deployment in other geographies.

In conclusion, our preliminary results show that the novel AI system has high sensitivity and specificity in detecting ROP. The next steps would be a prospective study to evaluate the integration of AI into the clinical workflow, and a comparison with the current standard of care to show reproducibility and consistency of the algorithm. We conclude that our findings are encouraging, and further work remains to provide more insight and understanding of the true potential of this technology. We acknowledge that the increased capacity to screen is only of value if there is scope to provide timely treatment. Advances in screening with technology such as this need to be met with increased treatment capacity, particularly for diseases such as ROP, given the time-criticality of interventions to prevent blindness. Hence, it is important that healthcare system strengthening for ROP management be started in parallel.

## Data Availability

The data analyzed in this study is subject to the following licenses/restrictions: Data could be made available upon reasonable request to the corresponding author for researchers who meet criteria to access confidential data bound by ethical restrictions. Requests to access these datasets should be directed to anandvinekar@yahoo.com.
